# Early initiation of breastfeeding, colostrum avoidance, and their associated factors among mothers with under one year old children in rural pastoralist communities of Afar, Northeast Ethiopia: a cross sectional study

**DOI:** 10.1186/s12884-020-03151-z

**Published:** 2020-08-05

**Authors:** Gebretsadkan Gebremedhin Gebretsadik, Helen Tkuwab, Kidanemaryam Berhe, Afework Mulugeta, Hajira Mohammed, Abebe Gebremariam

**Affiliations:** 1 Department of Nutrition and Dietetics, School of Public Health, College of Health Sciences, Mekelle University, Mekelle, Ethiopia; 2 Emory University, Addis Ababa, Ethiopia

**Keywords:** EIBF, Colostrum avoidance, Pastoralist, Ethiopia

## Abstract

**Background:**

Early initiation of breastfeeding (EIBF) is defined as initiation of breastfeeding within 1 h of birth. This is also the time colostrum is secreted with its potential benefits. Globally, two out of five under 5 children die in the first month of life, more than a third of which being on the first day. Neonatal mortality is still a major health problem in Ethiopia. EIBF and colostrum feeding are associated with decreased neonatal morbidity and mortality. With this study, we aim to determine the magnitude and factors associated with EIBF and colostrum avoidance.

**Methods:**

A community based cross-sectional study was conducted from May to June 2016 on 390 mothers in Afar region. Bivariate logistic regression was used to identify the association between the independent and the outcome variables. Multivariable logistic regression was used to determine the independent predictors of EIBF and colostrum avoidance. The strength of the association was measured by odds ratio and 95% confidence interval, and *p*-value < 0.05 was considered statistically significant. Hosmer and Lemeshow test was used to test model goodness of fitness and multi-collinearity between independent variables was checked.

**Results:**

About 248(63.6%) respondents initiated breastfeeding within 1 h of birth. Mothers whose delivery was attended by a health professional had 4.75 times higher odds (AOR 4.75; 95% CI 1.71, 13.19) of EIBF as compared to those who were attended by others. Trust on nurses to provide pregnancy care (AOR 5.59; 95% CI 1.05, 29.8) was significantly associated with EIBF. About 300(76.9%) respondents discarded colostrum. Mothers who had no discussion with TBA on child nutrition were 6.6 times (AOR 6.63; 95% CI 1.43, 30.63) more likely to avoid colostrum than their counterparts.

**Conclusion:**

More than one-third of infants didn’t start breastfeeding within 1 h of birth and three-fourth of the mothers discarded colostrum. Therefore, it is important to develop and/or strengthen services/advice on EIBF including colostrum feeding. Promoting delivery by health professionals, discussion on child nutrition and building trust between mothers and health professionals can be important community interventions to encourage EIBF and colostrum feeding.

## Background

Early (timely) initiation of breastfeeding (EIBF) is defined as the initiation of breastfeeding within 1 h of birth. This is also the time colostrum is secreted with its potential benefits [1]. The World Health Organization (WHO) and United Nations Children’s Fund (UNICEF) together recommend EIBF, exclusive breastfeeding for the first 6 months, and continued breastfeeding up to the age of 2 years and beyond, along with proper complementary feeding, as optimal breastfeeding practices [1].

Globally, only two out of five (42%) newborns start breastfeeding within the first hour of life. EIBF rates vary from 35% in the Middle East and North Africa to 65% in Eastern and Southern Africa [2]. EIBF, colostrum being the main player, is a key element of essential newborn care as it gives newborns the best chance to defend infections, survive, grow and develop to their full potential [3].

In 2015, about 2.7 million newborns died globally during the first month of life (0–27 days), more than a third of which being on the first day [4, 5]. Different studies have shown that delayed initiation of breastfeeding is associated with a higher risk of neonatal mortality. A systematic review described that initiation of breastfeeding after 1 h increases risk of neonatal mortality by 33% [6]. Another study also reported that about 22% of neonatal deaths could be prevented if breastfeeding is started within an hour of birth [7]. Besides, it has been shown that EIBF is a simple intervention with the potential to significantly improve neonatal outcomes and should be universally recommended, together with exclusive breastfeeding in the first month of life [8–10].

Colostrum is very vital for newborns in protecting infections particularly marked by its higher content immunoglobulins [11]. Together in, colostrum feeding showed association with lower odds of the three indicators of child undernutrition (stunting, underweight and wasting) [12].

In Ethiopia, neonatal mortality continues to be a major health problem. It accounts for 41% of under-five deaths [13]. Recent studies have also shown higher prevalence rates. The neonatal mortality rate was 18.6 and 20.2 per 1000 live births in studies done in Northern Ethiopia [14] and Arbaminch hospital [15], respectively. Besides, according to the 2019 Ethiopian mini-demographic health survey (EMDHS), 30 neonates out of 1000 live births die in the first month of life, which is not an improved number compared to the prevalence reported by 2016 EDHS (neonatal mortality was 29/1000 live births) [16]. This clearly indicates that neonatal mortality is not decreasing despite different interventions.

Ethiopia follows the WHO recommendations for the focused antenatal care (ANC) services. Pregnant mothers get such services at four different times either at health facilities or/and through home to home visit [16]. The attendants in health facilities include medical doctors, nurses, midwives, or/and health officers who have at least got a bachelor degree. The home to home services are often delivered by health extension workers or/and traditional birth attendants who haven’t got a bachelor degree.

In 2009, the WHO reported that about 69.1% of newborns in Ethiopia start to breastfeed within 1 h of birth [17]. Different studies have shown different levels of practice of EIBF. About 76.8, 83.7, 62.6, and 39.6% newborns were put to the breast within 1 h in Debre tabor [18], southern Ethiopia [19], Debre Berhan [20], and Amibara district [21], respectively. Additionally, different studies showed a higher prevalence of colostrum avoidance. About 25.6, 11.4, and 12% newborns didn’t feed colostrum in studies done in Debre Tabor [18], Kombolcha town [22], and North Wollo [23], respectively.

Although there are studies that assessed factors associated with EIBF and colostrum feeding, neonatal morbidity and mortality still remain major health concerns in the country. The aim of this study is to determine the prevalence and factors associated with EIBF and colostrum avoidance in Afar regional state, Ethiopia. Findings from this study might be used as inputs in developing local and national neonatal care guidelines, policymaking, and research so that EIBF and colostrum avoidance practices can be improved and potentially reduce neonatal mortality rate.

## Methods

### Study setting and design

This community-based cross-sectional study was conducted in Afar regional state, one of the regional states in Ethiopia, from May to June 2016. The region is classified under the desert and semi-desert agro-ecological zone and has a total surface area of 97, 256 sq. km with an altitude of 200 m below sea level to 1500 m above sea level. It is divided into 5 zones, 32 woredas (The second smallest administration units, 2 city administrations and 402 rural kebeles (The smallest administration units within woredas) with total population of 1.769 million projected for the year 2015. Women of reproductive age group and under-five children accounted for 22.8% (404,017) and 10.1% (178,972) of the total population, respectively, with an expected 50,857 live births per year.

### Sample size determination and sampling procedure

Our sample was drawn from a pre-intervention community-based newborn care (CBNC) quantitative data which was collected in zone one of Afar region. A total of 390 women of 15–49 years of age with live births and who had answered the outcome variables made up the sample size.

All woredas in zone one were purposively included as they are believed to be representatives of the whole context of Afar region with respect to the child and maternal health care and other characteristics. A two-stage cluster sampling technique was used to select sample clusters and households based on the estimated sample size. The primary sampling units (PSU) for the survey were kebeles which are located in the woredas and the secondary sampling units (SSU) were households. The total number of households with 0–11 months old children were chosen from each woreda as determined by proportion to population size (PPS) using the number of 0–11 months old children as the measure of size. The selection process for the kebeles (clusters) and households was random. In cases where the numbers of households with 0–11 months old children were small, a census was done to include all the mothers of the 0–11 months old children. Further information about the methodology of this study is published elsewhere [24] .

### Data collection tools and data quality assurance

Data were collected using an interviewer-administered questionnaire (Supplement 1). The questionnaire was translated to Amharic before the actual data collection. Data were collected by 12 trained data collectors under close follow-up of each and every activity of the data collection by six supervisors.

In order to assure the quality of data, prior to the actual surveys, the data collectors, supervisors, and coordinators were trained for 5 days on the objective of the study and data collection tools including demonstration/exercise of administration of questionnaires. Moreover, the questionnaires were tested in approximately 3% of the households to ensure that there are no errors in the questionnaire design and that the respondents can easily understand and respond to the questions. Filled-in questionnaires were checked for accuracy and completeness on a daily basis. If a problem or doubt arose, the data collectors were sent back to the household or health facility to rectify the problem. The quality of data was further ascertained during the data entry and cleaning process.

### Data analysis

Data were entered into and analyzed by Statistical Package for Social Sciences (SPSS) version 25. Descriptive statistical analysis was made to compute the mean, frequencies and percentages on selected variables. Binary logistic regression analysis was used to identify any association between the dependent and independent variables. The Crude Odds Ratios (COR) with a 95% confidence interval were estimated in the bivariate analysis to assess the association between each independent variable and the outcome variable. Variables with *p*-value < 0.3 in the bivariate logistic regression analysis were considered as candidates to be included in the multivariate logistic analysis.

In the multivariate analysis backward logistic regression method was used with the Hosmer-Lemeshow goodness-of-fit as a test for model fitness. Adjusted Odds Ratio (AOR) with 95% confidence interval was estimated to assess the strength of the association. There was no multicollinearity among the independent variables. A *p*-value of less than 0.05 was considered as a cut-off to detect a significant association between the independent and dependent variables. Findings are presented using textual descriptions, tables, and figures.

## Results

### Socio-demographic and economic characteristics

Three hundred eighty nine (99.7%) of the respondents were mothers. The median (±IQR) age of the mothers was 25 years (±10) and more than half (55.9%) of the respondents’ age was 25 years and below. The median age at first marriage of the mothers was 16 years (±2). Additionally, the median (±IQR) family size was 5 (±3) and almost all (99.2%) of the respondents were Muslim in religion. A majority (82.5%) of the spouses/husbands of the respondents didn’t attend any school for education. Fifty (12.9%) and 36 (9.3%) of the respondents had the ability to read and write and have attended formal schools for education, respectively. Three hundred fifty (91.4%) respondents had a monthly income of less than or equal to $31 (Table 1).

**Table 1:** Background characteristics of respondents in Afar Regional State, Ethiopia, 2016, *n* = 390

Variable (missing)	Frequency (%)
Ethnicity (0)	
Afar	380(97.4)
Amhara	9(2.3)
Oromo	1(0.3)
Total	390(100%)
Marital status (0)	
Married	387(99.2)
Separated	1(0.3)
Divorced	2(0.5)
Total	390(100%)
Educational status of respondents (0)	
Not attended formal education	354(90.8%)
Primary education	31(7.9%)
Secondary education	5(1.3%)
Total	390(100%)
Educational status of spouses (1)	
Not attended formal education	321(82.5%)
Primary education	40(10.3%)
Secondary education	17(4.6%)
College/university	7(1.8%)
Total	389(100%)
Presence of radio in the household (0)	
No	296(75.9)
Yes	94(24.1)
Total	390(100)

### Health services availability and utilization

The nearest health facility for 152 (65.1%) of the respondents was health post. One hundred forty-six (39.2%) respondents visited health posts during the last year. Half of the respondents were visited by health extension workers (HEWs) in the last year. Besides, half of these respondents always trust the pregnancy and delivery care provided by HEWs. More than one-third of the respondents trust HEWs to provide care during their pregnancy and birth. Similarly, more than half (57.5%) and 52.8% respondents always or sometimes trust nurses when providing them care during pregnancy and delivery, respectively.

One-third (34.8%) of the participants received advice from traditional birth attendants (TBA) but only 101(25.9%) reported discussions related to child nutrition. TBAs are individuals who have not received any formal midwifery training but attend births because the community believes they are capable of doing it. Around one-third of the respondents always trust TBA during pregnancy (30.8%) and delivery (32.1%) care. Twenty-six percent of the respondents spent more than an hour to reach health posts.

### Child health and care-seeking behavior

Only 29.7% of the respondents have antenatal care (ANC) visits to health institutions. Among these respondents, only one-third received information on nutrition. Besides, a similar number of respondents were advised on birth preparedness and complication readiness. Almost all (97.4%) respondents didn’t attend pregnant women conferences. Around one-third (33.5%) of the respondents made preparations for food and nutrition during delivery. As compared to the amount they usually take, very few (14%) respondents fed more during their pregnancy period. One-hundred four (80%) of those respondents who had ANC visits were fully satisfied with the pregnancy care. Around two-thirds (70.3%), 10.7, 8.2, 10.3% of the respondents didn’t have ANC visits, had 1–2 visits, had 3 visits, and had four or more ANC visits at health facilities in any health institution, respectively.

### Delivery and immediate newborn care

Majority (88.2%), 7, and 3.8% of the mothers delivered at home, at health center, and at public hospitals, respectively. The respondents reported various reasons for giving birth at home. The main reasons include not customary (in accordance with culture) to go to health facilities (39.5%), too far/no transportation (20.8%), traditional or religious reasons (14.1%), and thinking health facility is not necessary (4.1%). About 48(12.3%) respondents were fully satisfied during the delivery services. Immediately after delivery, 32(8.2%) babies were placed on the mother’s belly or chest, 30(7.7%) on the floor alone, and 315(81%) were placed beside the mother. Majority (92.3%) of the babies had no difficulty of crying/breathing at birth. Almost all (98.7%) respondents breast feed their babies. During delivery, Forty five (11.8%) respondents were assisted by skilled health professionals and the rest were assisted by traditional birth attendants, families, and friends. The respondents reported various reasons for giving birth at home.

### Early initiation of breastfeeding, colostrum avoidance, and their associated factors

Two hundred forty eight (63.6%) respondents initiated breastfeeding within 1 h of birth. Besides, 122(31.3%) started breastfeeding after an hour of birth but on the first day and 20(5.1%) started after the first day.

The variables that passed the screening (*p*-value< 0.3) in the bivariate analysis include husband’s attendance of formal school, ability of respondent to read and write, presence of health post in the kebele, respondent visited health post in the last year, time to reach health post, trust on nurses to provide pregnancy care, ANC visit during the last pregnancy, place of delivery, and delivery attendant.

The multivariate logistic analysis showed that trust on nurses to provide pregnancy care and delivery attendant were significantly associated with EIBF (*p*-value < 0.05) (Table 2). The odds of EIBF were 6 times (AOR 5.59; 95% CI 1.05, 29.8) higher in mothers who sometimes trust nurses to provide pregnancy care compared to those who have no trust. Additionally, mothers whose delivery was attended by a skilled attendant (qualified health care provider) showed higher odds (AOR 4.75; 95% CI 1.71, 13.19) of EIBF as compared to those who were attended by TBA, family, friends, or others during delivery. However, the remaining variables didn’t show significant association with the outcome variable.

**Table 2:** Bivariate and multivariate logistic regression analysis of independent variables for early initiation of breastfeeding in Afar Regional State, Ethiopia, 2016

Variables	Timely breastfeeding initiation		COR (95%CI)	AOR(95%CI)
	No	Yes		
Husband attended any school				
No	123	198	0.62(0.35,1.11)	1.09(0.39,3.10)
Yes (ref.)	19	49	1	1
Total	142	247		
Respondent able to read and write				
No	128	210	0.52(0.26,1.03)	0.61(0.228,1.61)
Yes (ref.)	12	38	1	1
Total	140	248		
Presence of health post in the kebele				
No	32	75	1.47(0.91,2.38)	1.92(0.14,26.84)
Yes (ref.)	103	164	1	1
Total	135	239		
Time to reach health post				
< =30 min	72	95	0.49(0.266,0.92)	0.56(0.24,1.29)
30–60 min	6	12	0.75(0.25,2.29)	0.51(0.13,1.99)
> 60 min(ref.)	18	48	1	1
Total	96	155		
Respondent visited HP during the last year				
No	72	155	1.48(0.96,2.28)	1.83(0.92,3.61)
Yes (ref.)	59	86	1	1
Total	131	241		
Trust nurses on providing pregnancy care				
I always trust them	48	58	0.40(0.23,0.69)	2.98(0.43,20.82)
I sometimes trust them	23	46	0.67(0.35,1.26)	5.59(1.05,29.81)*
I don’t trust them(ref.)	33	99	1	1
Total	104	203		
ANC visit in the last pregnancy				
No	102	172	0.89(0.56,1.40)	1.12(0.29,4.37)
Yes (ref.)	40	76	1	1
Total	142	248		
Place of delivery				
At home	131	214	0.53(0.26,1.08)	0.59(0.16, 2.27)
At health institution (ref.)	11	34	1	1
Total	142	248		
Delivery attended by who				
Skilled attendant	11	36	2.03(0.99,4.14)	4.75(1.71,13.19)*
Other (ref.)	128	206	1	1
Total	139	242		

*=*p*-value< 0.05, *CI* Confidence Interval, *COR* Crude Odds Ratio, *AOR* Adjusted Odds Ratio Hosmer-Lemeshow goodness-of-fit = 0.814

Furthermore, about 300(76.9%) respondents avoided the first milk (colostrum). Six variables (Respondent able to read and write, discussion with TBA on child nutrition, number of live births, ANC visit during the last pregnancy, counseling received on newborn care, and presence of health post in the kebele) were included in the final multivariable logistic regression analysis after passing the screening through bivariate analysis (*p*-value< 0.3).

The multivariate logistic regression analysis showed that only one variable is significantly associated with colostrum avoidance at *p*-value < 0.05. Mothers who had no discussion with TBA on child nutrition were about 6.6 times (AOR 6.63; 95% CI 1.43, 30.63) more likely to avoid colostrum than those who made discussion with TBA on child nutrition. (Table 3).

**Table 3:** Bivariate and multivariate logistic regression analysis of independent variables for colostrum avoidance in Afar Regional State, Ethiopia, 2016

Variables	Colostrum avoided		COR (95%CI)	AOR(95%CI)
	No	Yes		
Respondent able to read and write				
No	83	255	0.5(0.22,1.54)	0.48(0.03,8.17)
Yes (ref.)	7	43	1	1
Total	90	289		
Discussion with TBA on child nutrition				
No	13	88	5.92(1.84.19.08)	6.63(1.43,30.63)*
Yes (ref.)	7	8	1	
Total	20	96		
Number of live births				
1	19	43	0.62(0.34,1.13)	0.61(0.09,4.4)
> 1 (ref.)	70	255	1	1
Total	89	298		
ANC visit in the last pregnancy				
No	70	204	0.61(0.35,1.06)	1.12(0.29,4.37)
Yes (ref.)	20	96	1	
Total	90	300		
Counseling received on new born care				
No	84	268	0.52(0.19,1.37)	0.42(0.05,4.03)
Yes (ref.)	5	31	1	
Total	89	299		
Presence of health post in the kebele				
No	32	74	0.52(0.31,0.87)	0.26(0.07,1.02)
Yes (ref.)	49	219	1	
Total	81	293		

*=*p*-value< 0.05, *CI* Confidence Interval, *COR* Crude Odds Ratio, *AOR* Adjusted Odds Ratio

## Discussion

Timely initiation of breastfeeding, avoidance of colostrum and associated factors were assessed in Afar Regional State. EIBF is breastfeeding within 1 h of birth. Delayed breastfeeding initiation means initiating breastfeeding after an hour after birth [1]. EIBF is important for both the health of the child and the mother and it increases the bond between the baby and the mother [25, 26]. But in this study, only 63.6% of the children initiated breastfeeding within the first hour after birth.

A meta-analysis on prevalence of key breastfeeding indicators was conducted in 29 sub-Saharan African countries. The prevalence of EIBF in this study was found within the range of the finding in the 29 sub-Saharan African countries which was between 37.8 and 69.3% [27] but above the prevalence found in the low and middle income countries (31–60%) [28] and world (57.6%) [29].

The prevalence of delayed initiation of breastfeeding in this study was lower compared to prevalence reported from studies conducted in Gurage Zone, Hula District, South Gondar Zone, Goba Woreda, Jimma Arjo Woreda, Two Agro-ecological Zones, Sudan, India, Bangladesh, and Nepal which was 52.7, 49.4, 49.3, 47.6, 40.3, 37, 52, 43.5, 48.6, 58.2%, respectively [30–39]. The variation could be due to a difference in study period, health policy, and socio-economics characteristics. In contrast, the prevalence in this study was higher as compared to the prevalence reported from studies conducted in East Wolega Zones, Bahir Dar City, Dembecha district, and Uganda which was 16.9, 24.6, 26.9, and 31.4%, respectively [40–43]. The difference might be due to differences in access to health information, socio-economic status, infrastructure, educational status, cross-cultural differences in breastfeeding practice, and health service access and utilization characteristics.

In this study, associated factors for EIBF were identified. Mothers who trusted nurses in providing care during pregnancy had higher odds of initiating breastfeeding within the first hour after delivery [5.59, 95%CI (1.05, 29.81)]. The counseling and information given on breastfeeding by nurses can encourage mothers to initiate breastfeeding early [44]. This study also showed that mothers who delivered with assistance of health professionals initiated breastfeeding within the first hour of delivery as compared to mothers with no assistance at the time of delivery [4.75, 95% CI (1.71, 13.19)]. This could be due to the support and counseling health professionals provide to mothers to initiate breastfeeding within the first hour of birth [2, 44]. This finding was also consistent with findings from another study [45].

Colostrum is the first breast milk that contains antibodies that can protect the newborn from diseases [26]. However, this study revealed that 76.9% of the mothers discard the colostrum. This finding is higher compared to findings reported from studies in Raya Kobo district (13.5%), Axum (6.3%), Bahir Dar City (23.8%), Debre Markos Town (66.5%), rural India (16.7%), and Bangladesh (54%) [46–51]. The possible explanations for this difference could be the differences in socioeconomic status, differences in access and use of health services, and the fact that this study was conducted in remote area that contains pastoral and agro-pastoral communities. Respondents who didn’t discuss on child nutrition with TBA were 6 times more likely to discard colostrum as compared to their counterparts [6.63, 95%CI (1.43, 30.63)]. This study has certain limitations. One limitation is the possibility to have recall bias. Secondly, due to the nature of the study design i.e. cross-sectional, it is difficult to establish cause-effect relationship. Lastly, even though we tried to control for the effect of possible confounders during data analysis, we can’t completely rule out the possible effect of confounders.

## Conclusion

Delayed initiation of breastfeeding was common among mothers with under 1 year old children in rural pastoralist communities of Afar. Three-fourth of the mothers discarded colostrum. Trusting nurses on providing care during pregnancy and health profession delivery assistance were positively associated with EIBF. The only associated factor for colostrum avoidance was having no discussion with TBA on child nutrition. It is important to develop and/or strengthen services/advice on early initiation of breastfeeding including colostrum consumption. Promoting delivery by health professions, discussion on child nutrition and building trust between the mothers and health professions can encourage EIBF and colostrum feeding.

## Supplementary information

**Additional file 1.**

## Data Availability

The datasets used and/or analyzed during the current study are available from the corresponding author on reasonable request.

## Abbreviations

ANC: Ante-Natal Care
AOR: Adjusted Odds Ratio
CI: Confidence interval
COR: Crude Odds Ratio
EDHS: Ethiopian Demographic and Health Survey
EIBF: Early Initiation of Breast Feeding
HEW: Health Extension Workers
IQR: Inter-Quartile Range
OR: Odds Ratio
SPSS: Statistical Package for Social Sciences
TBA: Traditional Birth Attendants
UNICEF: United Nations International Children’s Emergency Fund
WHO: World Health Organization
