# Supplementary material for: Early initiation of breastfeeding, colostrum avoidance, and their associated factors among mothers with under one year old children in rural pastoralist communities of Afar, Northeast Ethiopia: a cross sectional study
Source: BMC Pregnancy Childbirth. 2020 Aug 5;20:448. doi: 10.1186/s12884-020-03151-z (PMC7405449; doi:10.1186/s12884-020-03151-z)
Supplement: Supplementary file 1 — Additional file 1. [file 12884_2020_3151_MOESM1_ESM.doc]

**
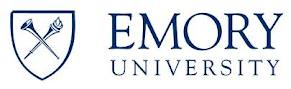
**

**Emory University, ICCM/CBNC Afar Project**

**CBNC Household Survey Questionnaire V 1.0**

| **Module II: CBNC Questionnaire** | | | |
| --- | --- | --- | --- |
| **Section I: IDENTIFICATION AND CONSENT**   | R | Z | W | K | G | HH | | --- | --- | --- | --- | --- | --- | |  |  |  |  |  |  | | | | |
| **Informed consent obtained:** **1 = Yes 2 = No** | | | |
| **101**  **102**  **103**  **104**  **105**  **106**  **107**  **108**  **109**  **110**  **111**  **112**  **113**  **114**  **115**  **116**  **117** | **INTERVIEWER COMPLETE:**  Interviewer Number: ________________________________________ [INT_NUM]  Interviewer Name: ________________________________________  Survey Identification Number: ________________________________________________ [ID]  Zone: (WRITE IN SPACE)____________________________________________________ [ZONE]  Zone ID: (CHECK CODEBOOK:1-2) _______ [ZONEN]  Woreda: (WRITE IN SPACE)____________________________________________________ [WOREDA]  Woreda ID: (CHECK CODEBOOK:1-13) _______ [WOREDAN]  PHCU HC name: (WRITE IN SPACE )_______ [PHCU]  PHCU Cluster ID: (CHECK CODEBOOK: 1-20)_______ [PHCUN]  Kebele: (WRITE IN SPACE)_____________________________________________________[KEBELE]  Kebele ID: (CHECK CODEBOOK: 1-40)_______ [KEBELEN]  Gote: (WRITE IN SPACE)_____________________________________________________[GOTE]  Household ID: (WRITE IN SPACE)______________________________________________[HHN]  Date Interview Performed: _________________________________  Date Checked by supervisor__________________________________  Date Checked by coordinator _____________________________  Name of household head _____________________________ | | |
| **118** | Result Codes: | 1 = Completed  2 = Partly completed  3 = Postponed  4 = Not at home  5 = No-one competent to respond |  |

| **Section II: BACKGROUND AND HOUSEHOLD CHARACHTERISTICS** | | | | | |
| --- | --- | --- | --- | --- | --- |
| **Interview Say*: “I am going to start by asking you some questions about you and your household.”***  **Interviewer: In this section and throughout the questionnaire, please read out all the options to the interviewee UNLESS it is clearly stated ‘Do not read list’** | | | | | |
| **201** | How old are you? | [_____] in completed years  99 = Don’t Know | | |  |
| **202** | How long have you been living continuously in (NAME OF CURRENT PLACE OF RESIDENCE)?  IF LESS THAN ONE YEAR, RECORD '00' YEARS. | [_________] years [_________] months  95 = Always  96 = Visitor | | |  |
| **203** | What is your religion? | 1 = Muslim  2 = Christian orthodox  3 = Christian protestant  4 = Christian (other)  5 = Traditional  88 = Other(Specify)_______________ | | |  |
| **204** | What is your ethnicity? | 1 = Afar  2 = Amhara  3 = Tigre  4 = Oromo  88 = Other(Specify)_______________ | | |  |
| **205** | What is your current marital status? | 1 = Married  2 = Live with cohabit  3 = Separated  4 = Divorced  5 = Widowed  6 = Single | | | **2/6🡪207** |
| **206** | How old were you when you were married, first? | [________] years  99 = Don’t Know | | |  |
| **207** | Did your spouse/husband attend any school? | 1 = Yes  2 = No | | | **🡪209** |
| **208** | Educational status – Spouse (If attended formal education write the highest grade completed) | Grade [___|___]  13 = Technical/vocational certificate  14 = University/college diploma  15 = University/college BSC or Higher | | |  |
| **209** | Are you able to read and write? | 1 = Yes  2 = No | | | **2🡪212** |
| **210** | Did you ever attend formal school? | 1 = Yes  2 = No | | | **2🡪212** |
| **211** | If yes Q. 210, educational status - the highest grade you completed? | Grade [___|___]  13 = Technical/vocational certificate  14 = University/college diploma  15 = University/college BSC or Higher | | |  |
| **212** | How many people reside in this household? | [________] | | |  |
| **213** | Did you earn any cash income during last month? | 1 = Yes  2 = No | | |  |
| **213** | Did your spouse or husband earn any cash income during the last month? | 1 = Yes  2 = No | | |  |
| **214** | What is the total amount of income your families earned during last month?  **If No income during last month insert “00”** | [________] | | |  |
| **215** | Main material of the household floor. **Record observation.** | 1 = Natural floor (earth/sand/dung)  2 = Rudimentary floor (wood/palm/bamboo)  3 = Finished floor (polished wood/ vinyl/tiles/cement/carpet)  88 = Other (Specify)___________________ | | |  |
| **216** | Main material of the household roof. **Record observation** | 1 = Thatch/grass or leaves  2 = Iron sheets or tiles  88 = Other (Specify)___________________ | | |  |
| **217** | Main material of the household walls. **Record Observation** | 1 = No walls  2 = Natural materials (cane, wood, mud, straw)  3 = Stone with mud  4 = Stone/bricks with cement  88 = Other (Specify)___________________ | | |  |
| **218** | How many rooms in this household are used for sleeping? | Number of rooms [ _____________ ] | | |  |
| **219** | What is the main source of drinking water for members of your household? | 1 = Piped into dwelling  2 = Piped into compound  3 = Piped outside compound  4 = Covered Well  5 = Protected Spring  6 = Open Well  7 = Open Spring  8 = River  9 = Pond/Lake/Dam  10 = Rainwater  88 = Other (Specify)____________________ | | |  |
| **220** | How long does it take you to go there, get water and come back? | Minutes [____|____]  Hours [____|____]  96 = On premises | | |  |
| **221** | Do you treat your water in any way to make it safer to drink? | 1 = Yes  2 = No | | | **2 🡪223** |
| **222** | What do you usually do to the water to make is safer to drink? | 1 = Boil  2 = Use wuha-agar  3 = Add bleach/chlorine  4 = Strain it through a clot  5 = Use water filter *(ceramic, sand composite)*  6 = Solar disinfection  7 = Let it stand and settle  88 = Other (specify)________________  99 = Do not Know | | |  |
| **223** | What kind of toilet facility does most members of your household use? | 1 = Pit Latrine/traditional pit toilet  2 = Ventilated improved pit latrine (VIP)  3 = Flush toilet  4 = No facility/Bush/Field  88 = Other(Specify)____________________ | | |  |
| **224** | The last time you passed stool, where did you use/defecate? | 1 = Pit Latrine/traditional pit toilet  2 = Ventilated improved pit latrine (VIP)  3 = Flush toilet  4 = No facility/Bush/Field  88 = Other(Specify)____________________ | | |  |
| **225** | What type of fuel does your household mostly use for cooking?  **Do not read list** | 1 = Dung  2 = Firewood/straw  3 = Charcoal  4 = Kerosene  5 = Gas  6 = Electricity  88 = Other (specify) ____________________ | | |  |
| **226** | Does your household have: | **Variables** | **Yes** | **No** |  |
|  |  | Electricity? | 1 | 2 |  |
|  |  | Watch/clock? | 1 | 2 |  |
|  |  | Radio? | 1 | 2 |  |
|  |  | A television? | 1 | 2 |  |
|  |  | Mobile telephone? | 1 | 2 |  |
|  |  | Non-mobile telephone? | 1 | 2 |  |
|  |  | A refrigerator? | 1 | 2 |  |
|  |  | Table? A chair? | 1 | 2 |  |
|  |  | bed with cotton/sponge mattress? | 1 | 2 |  |
|  |  | An electric mitad? | 1 | 2 |  |
|  |  | Kerosene Stove? | 1 | 2 |  |
|  |  | Kerosene lamp/pressure lamp? | 1 | 2 |  |
|  |  | Gun? | 1 | 2 |  |
|  |  | Kitchen? | 1 | 2 |  |
|  |  | A bicycle? | 1 | 2 |  |
|  |  | Motorcycle or motor scooter/Bajaj? | 1 | 2 |  |
|  |  | An animal-drawn cart? | 1 | 2 |  |
|  |  | A car or truck? | 1 | 2 |  |
| **227** | Does this household own any livestock, herds, or farm animals? | 1 = Yes  2 = No | | | **2 🡪229** |
| **228** | If yes Q 227, how many of the following animals, does this household own? If none record “00” | [_______] Milk cows, oxen  [_______] Horses  [_______] Donkeys  [_______] Mules  [_______] Goats  [_______] Sheep  [_______] Chickens  [_______] Camel  [_______] Other _________________ | | |  |
| **229** | Do you have (LOCAL UNITS) of agricultural land that this household member own? | 1 = Yes  2 = No | | | **2 🡪301** |
| **230** | If yes, Q. 229, how many (LOCAL UNITS) of agricultural land do you own?  If unknown, record “999” | Local Units (Timad/hectar/other unit) .. [ ] | | |  |

| **Section III: BIRTH HISTORY** | | | |
| --- | --- | --- | --- |
| **Interview Say***: “Now I would like to ask you some questions about your experiences with pregnancy and childbirth.”*  **Interviewer: write name of woman: [____________________________________]**  **Interviewer: write woman ID: [_______________]** | | | |
|  | How many times have you **been pregnant**? | [_____] times  99 = Don’t Know |  |
|  | How many **live births** have you had in your life time? | [_____] times  99 = Don’t Know |  |
|  | How many living sons and daughters do you have who are < 5 years old? | [_____] sons < 5 years AND  [_____] daughters <5 years  [_____] total number of < 5 years |  |
|  | Have you ever had a pregnancy that didn’t end up in a live birth? | 1 = Yes  2 = No | **2 🡪306** |
|  | If yes **Q. 303**, how many pregnancies have you had that didn’t end up in live birth? | [_____] pregnancies |  |
|  | How many pregnancies lost before 7 months (28 weeks)? | [_____] pregnancies |  |
|  | Have you ever had a child who died in the first year of his/her life? | 1 = Yes  2 = No | **2 🡪310** |
|  | Were any of the children who died in the ***first 28 days*** of life? | 1 = Yes  2 = No |  |
|  | Were any of the children who died in the ***first seven days*** of his/her life? | 1 = Yes  2 = No |  |
|  | Were any of the children who died in the ***first 48 hours*** of his/her life? | 1 = Yes  2 = No |  |
|  | Are you currently pregnant or intending to become pregnant? | 1 = Yes  2 = No | **1 🡪400** |
|  | If no for **Q. 310**, are you currently using a family planning method to avoid or delay pregnancy? | 1 = Yes  2 = No | **2 🡪400** |
|  | Which of the following family planning method are you using currently? | 1 = Oral contraceptive Pills  2 = Condom (male)  3 = Condom (female)  4 = Male sterilization  5 = Female sterilization  6 = IUD  7 = Rhythm method  8 = Abstinence from sex  9 = Implants  10 = Injectable (DEPO)  11 = Diaphragm  12 = Withdrawal method  13 = Lactational amenorrhea /Breast feeding  14 = Others (Specify) __________________ |  |
|  | Do you know of a place where you can obtain a family planning method? | 1 = Yes  2 = No |  |
|  | Where can you obtain a family planning method? | 1 = Health Post  2 = Health Center  3 = Government Hospital  4 = Non-Governmental clinic  5 = Private Hospital  6 = Private clinic  7 = Private pharmacy  8 = Community based outlets  9 = Rural drug vendor  10 = Traditional practitioner/holy place  11 = Shop  12 = Other (specify) ____________________ |  |

| **Section IV: Access to Health Facility** | | | |
| --- | --- | --- | --- |
| **Interviewer Say***: “Now I would like to ask you some questions about your access to health facility and your trust for health care providers and community volunteers.”* | | | |
|  | Does the kebele have health facility? | 1 = Yes  2 = No  99 = Don’t know |  |
|  | How long does it take you to walk to the nearest health facility?  [If less than an hour, record it in Minutes] | Health post: Minutes [__|___] Hours [__|__]  Health Center: Minutes [__|_ _] Hours [__|__]  Other: Minutes [__|_ _] Hours [__|__] |  |
|  | What type of facility is this (the nearest)? | 1 = Health Post  2 = Health Center  3 = Government Hospital  4 = Non-Governmental clinic  5 = Private Hospital  6 = Private clinic  7 = Other (specify) ____________________  99 = Don’t Know |  |
|  | Does the kebele have health post? | 1 = Yes  2 = No  99 = Don’t know |  |
|  | Have you visited the health post at any time during last year? | 1 = Yes  2 = No  8 = No health facility access | **2🡪407**  **8🡪407** |
|  | When was the last time you visited the health post? | 1 = Within last 1 month  2 = With last 2-3 months  3 = Within 4-6 months  4 = > 6 months ago |  |
|  | The last time you visited the health post, what was the reason for you to visit the health post?  **Mentioned (M); Not Mentioned (NM)**  **Do not read the responses**  **(Multiple Responses Possible)** | M NM  Family planning ………………………1 2  Child immunization …………………1 2  Antenatal care ……………..…………1 2  Postnatal care………………….………1 2  Health education………………..……1 2  Growth monitoring………………..…1 2  Referral of sick child……………….…1 2  Diarrhea treatment…………………..1 2  Malaria treatment……………….……1 2  Pneumonia treatment………………1 2  receive bed nets…………………….…1 2  Delivery care……………………..………1 2  Neonatal care…………………….………1 2  Other, specify_____________________ |  |
|  | Have you heard of or do you know about the health extension worker? | 1 = Yes  2 = No | **2 🡪414** |
|  | Did they visit your house? | 1 = Yes  2 = No | **2 🡪410** |
|  | When was the last time she/he visited you? | 1 = Within last month  2 = Within last 2 – 3 months  3 = Greater than 3 months ago |  |
|  | Have you heard of or do you know about the health extension worker? | 1 = Yes  2 = No |  |
|  | How much do you trust a ***HEW*** to provide care while you are pregnant? | 1 = I always trust them  2 = I sometimes trust them  3 = I don’t trust them |  |
|  | How much do you trust a ***HEW*** to provide care while you are giving birth? | 1 = I always trust them  2 = I sometimes trust them  3 = I don’t trust them |  |
|  | How much do you trust a ***HEW*** to provide care after you have given birth? | 1 = I always trust them  2 = I sometimes trust them  3 = I don’t trust them |  |
|  | How much do you trust a ***HEW*** to provide care for your newborn baby? | 1 = I always trust them  2 = I sometimes trust them  3 = I don’t trust them |  |
|  | Have you heard of or do you know about the Frontline Workers? | 1 = Yes  2 = No | **2 🡪421** |
|  | Did they visit your house? | 1 = Yes  2 = No | **2 🡪417** |
|  | If yes **Q. 415**, When was the last time she/he visited you? | 1 = Within last month  2 = Within last 2 – 3 months  3 = Greater than 3 months ago |  |
|  | How much do you trust a ***FLW*** to provide care while you are pregnant? | 1 = I always trust them  2 = I sometimes trust them  3 = I don’t trust them |  |
|  | How much do you trust a ***FLW*** to provide care while you are giving birth? | 1 = I always trust them  2 = I sometimes trust them  3 = I don’t trust them |  |
|  | How much do you trust a ***FLW*** to provide care after you have given birth? | 1 = I always trust them  2 = I sometimes trust them  3 = I don’t trust them |  |
|  | How much do you trust a ***FLW*** to provide care for your newborn baby? | 1 = I always trust them  2 = I sometimes trust them  3 = I don’t trust them |  |
|  | How much do you trust a ***nurse*** at the health post to provide care while you are pregnant? | 1 = I always trust them  2 = I sometimes trust them  3 = I don’t trust them |  |
|  | How much do you trust a ***nurse*** at the health post to provide care while you are giving birth? | 1 = I always trust them  2 = I sometimes trust them  3 = I don’t trust them |  |
|  | How much do you trust a ***nurse*** at the health post to provide care after you have given birth? | 1 = I always trust them  2 = I sometimes trust them  3 = I don’t trust them |  |
|  | How much do you trust a ***nurse*** at the health post to provide care for your newborn baby? | 1 = I always trust them  2 = I sometimes trust them  3 = I don’t trust them |  |
|  | How much do you trust ***Voluntary Community Health workers*** to provide care while you are pregnant? | 1 = I always trust them  2 = I sometimes trust them  3 = I don’t trust them |  |
|  | How much do you trust ***Voluntary Community Health workers*** to provide care while you are giving birth? | 1 = I always trust them  2 = I sometimes trust them  3 = I don’t trust them |  |
|  | How much do you trust ***Voluntary Community Health workers*** to provide care after you have given birth? | 1 = I always trust them  2 = I sometimes trust them  3 = I don’t trust them |  |
|  | How much do you trust ***Voluntary Community Health workers*** to provide care for your newborn baby? | 1 = I always trust them  2 = I sometimes trust them  3 = I don’t trust them |  |
|  | Have you ever received service or advice from Traditional birth Attendants? | 1 = Yes  2 = No | **2 🡪431** |
|  | What was discussed or what services were provided by the Traditional Birth Attendants the last time he/she visited you at your home?  **Mentioned (M); Not Mentioned (NM)**    **Do not read the responses**  **(Multiple Responses Possible)** | Advice or information on M NM  Immunization……………………………..…….1 2  Child nutrition………………………………..….1 2  Diarrhea treatment…………………………...1 2  Care seeking for sick child………………....1 2  Pregnancy care/ANC…………….……..…….1 2  HIV/AIDS ………………………………..……..….1 2  Hygiene…………………………………………..….1 2  Promotion pit latrine construction..…...1 2  Promotion on latrine use…………….……..1 2  Promotion on safe water use….……..….1 2  Family planning ………………………………….1 2  Family health services…………………….….1 2  Do not know………………………..…………….8  Other, specify__________________ |  |
|  | How much do you trust a ***Traditional Birth Attendants*** to provide care while you are pregnant? | 1 = I always trust them  2 = I sometimes trust them  3 = I don’t trust them |  |
|  | How much do you trust a ***Traditional Birth Attendants*** to provide care while you are giving birth? | 1 = I always trust them  2 = I sometimes trust them  3 = I don’t trust them |  |
|  | How much do you trust a ***Traditional Birth Attendants*** to provide care after you have given birth? | 1 = I always trust them  2 = I sometimes trust them  3 = I don’t trust them |  |
|  | How much do you trust a ***Traditional Birth Attendants*** to provide care for your newborn baby? | 1 = I always trust them  2 = I sometimes trust them  3 = I don’t trust them |  |
|  | Have you heard of or do you know about social mobilization committee (Health Development Army) in your community? | 1 = Yes  2 = No | **2 🡪500** |
|  | Are you a member of SMC /HDA in your kebele? | 1 = Yes  2 = No |  |
|  | Did any member of SMC /HDA visit you in the home to talk about health related issues during the last six months? | 1 = Yes  2 = No | **2 🡪500** |
|  | What was the information /advise were provided by the SMC /HDA the last time he/she visited you at your home?  **Mentioned (M); Not Mentioned (NM)**  **Do not read the responses**  **(Multiple Responses Possible)** | Advice or information on M NM  Immunization………………………..……….….1 2  Child nutrition…………………………..….…….1 2  Diarrhea treatment……………………..…….1 2  Care seeking for sick child………….….…..1 2  Pregnancy identification..…….….…….….1 2  Pregnancy care/ANC…………….….…….….1 2  Skill delivery ……………………………………….1 2  Early PNC and notification ……………..….1 2  HIV/AIDS ………………………………….……….1 2  Hygiene……………………………………..……….1 2  Promotion pit latrine construction….….1 2  Promotion on latrine use…………….….….1 2  Promotion on safe water use….………….1 2  Family planning ………………………………….1 2  Do not know………………………..…………….99  Other, specify__________________ |  |

| **Section V: Antenatal Care (ANC)** | | | | | |
| --- | --- | --- | --- | --- | --- |
| **Interviewer: *Now I want to talk to you about your pregnancy with the birth of [Child name / pregnancy number]*  please use the pregnancy number to refer to the birth (instead of baby name)** | | | | | |
|  | When pregnant with CHILD NAME / PREGNANCY NUMBER, did you inform anyone outside your family? | 1 = Yes  2 = No | | | **2🡪503** |
|  | If yes Q.500, who was the first non-family member you told that you were pregnant? | 1 = HEW  2 = FLW  3 = Nurse  4 = TBA  5 = vCHW(HDA)  6 = Neighbor  7 = Traditional healers  8 = Religious leader  88 = Others (specify) __________________ | | |  |
|  | What was the duration of your pregnancy when you informed the person that you were pregnant? | [____] weeks  OR  [___] months  OR  99 = Don’t Know | | |  |
|  | Do you have a family health card (FHC) with information about that pregnancy and birth? | 1 = Yes  2 = No | | |  |
|  | When pregnant with [CHILD NAME / PREGNANCY NUMBER], did anyone advise you on the importance of care during pregnancy (ANC pregnancy checkups)? | 1 = Yes  2 = No | | | **2🡪506** |
|  | From whom did you first hear about the need to seek /Importance of antenatal care? | 1 = HEW  2 = FLW  3 = Nurse  4 = TBA  5 = vCHW(HDA)  6 = Relative/friend/Neighbor  7 = social gathering  8 = Religious leader  9 = Other family members  10 = Personal past experience  88 = Others (specify) __________________  99 = Don’t know | | |  |
|  | When pregnant with [CHILD NAME / PREGNANCY NUMBER], did you receive any care during pregnancy? | 1 = Yes  2 = No | | | **2🡪601** |
|  | If yes **Q. 506**, where did you get ANC visits?  **Multiple response** | 1 = Health Post  2 = HEW at home  3 = Health Center  4 = Government Hospital  5 = Private Hospital  6 = Private clinic  7 = At home by TBA/vCHW  88 = Other (specify) ____________________  99 = Don’t Know | | |  |
|  | How old was your pregnancy at the first visit? | [____] weeks  OR  [___] months  OR  99 = Don’t Know | | |  |
|  | How many times did you visit the health facility for pregnancy care (ANC) in your last pregnancy? | 1 = Only a visit  2 = Two visits  3 = Three visits  4 = 4+ visits  99 = Don’t know | | |  |
|  | Where did you get your first ANC visit? | 1 = Health Post  2 = HEW at home  3 = Health Center  4 = Government Hospital  5 = Private Hospital  6 = Private clinic  7 = At home by TBA/vCHW  88 = Other (specify) ____________________  99 = Don’t Know | | |  |
|  | Who attended you at the first visit of your last pregnancy? | 1 = Skilled health care provider  2 = FLW  3 = HEW  4 = TBA  5 = vCHW(HDA)  6 = Relative/friend/Neighbor  88 = Others (specify) ____________________  99 = Don’t know | | |  |
|  | ***During your ANC visit*** |  | | |  |
|  | Was your weight measured? | 1 = Yes  2 = No | | | **2🡪515** |
|  | Where was this service provided? | 1 = Health Post  2 = Health Center  3 = Government Hospital  4 = Private Hospital/clinic  5 = At home  88 = Other (specify) ____________________ | | |  |
|  | Which was the provider who measured your weight the first time?  Put 99 if not known | 1= Skilled Health care provider  2 = FLW  3 = HEW  4 = TBA  5 = vCHW(HDA)  88 = Others (specify) ____________  99 = Don’t know | | |  |
|  | Was your blood pressure measured? | 1 = Yes  2 = No | | | **2🡪518** |
|  | Where was this service provided? | 1 = Health Post  2 = Health Center  3 = Government Hospital  4 = Private Hospital/clinic  5 = At home  88 = Other (specify) ____________________ | | |  |
|  | Which was the provider who measured your blood pressure the first time?  Put 99 if not known | 1= Skilled Health care provider  2 = FLW  3 = HEW  4 = TBA  5 = vCHW(HDA)  88 = Others (specify) ____________  99 = Don’t know | | |  |
|  | Was your blood tested? | 1 = Yes  2 = No | | | **2🡪521** |
|  | Where was this service provided? | 1 = Health Post  2 = Health Center  3 = Government Hospital  4 = Private Hospital/clinic  5 = At home  88 = Other (specify) ____________________ | | |  |
|  | Which was the provider who prescribed you blood test the first time?  Put 99 if not known | 1= Skilled Health care provider  2 = FLW  3 = HEW  4 = TBA  5 = vCHW(HDA)  88 = Others (specify) ____________  99 = Don’t know | | |  |
|  | Was your urine tested? | 1 = Yes  2 = No | | | **2🡪524** |
|  | Where was this service provided? | 1 = Health Post  2 = Health Center  3 = Government Hospital  4 = Private Hospital/clinic  5 = At home  88 = Other (specify) ____________________ | | |  |
|  | Which was the provider who prescribed you urine test the first time?  Put 99 if not known | 1= Skilled Health care provider  2 = FLW  3 = HEW  4 = TBA  5 = vCHW(HDA)  88 = Others (specify) ____________  99 = Don’t know | | |  |
|  | Did you receive iron folate tablets? | 1 = Yes  2 = No | | | **2🡪533** |
|  | Where was this service provided? | 1 = Health Post  2 = Health Center  3 = Government Hospital  4 = Private Hospital/clinic  5 = At home  88 = Other (specify) ____________________ | | |  |
|  | Which was the provider who gave you iron folate tablet the first time?  Put 99 if not known | 1= Skilled Health care provider  2 = FLW  3 = HEW  4 = TBA  5 = vCHW(HDA)  88 = Others (specify) ____________  99 = Don’t know | | |  |
|  | For how many days/months did you take the tablets? | [____] days  OR  [___] months | | |  |
|  | Did the health care provider or other advise/counsel you about iron supplement? | 1 = Yes  2 = No | | | **2🡪530** |
|  | If yes **Q. 528**, what were the issues addressed during counseling? | 1 = Dose – daily dose  2 = Duration  3 = Benefits  4 = Side effects  5 = Ways to manage side effect  88 = Others (specify)___________________ | | |  |
|  | What benefits of iron folate do you know? | 1 = Prevents anemia  2 = Prevents dizziness  3 = Increases blood  4 = Increases energy  5 = prevent death  88 = Other (Specify)____________________  99 = Don’t Know | | |  |
|  | Did you experience any side effects while you took iron tablets? | 1 = Yes  2 = No | | | **2🡪533** |
|  | If yes **Q. 531**, what specific side effect you had encountered?  multiple responsible possible)  Don’t read the choices | 1 = Constipation  2 = Heart burn/epigastric pain  3 = Nausea /Vomiting  4 = Darkening of stool  5 = Other (specify) _____________________ | | |  |
|  | Did you receive any drug for intestinal worms? | 1 = Yes  2 = No | | | **2🡪536** |
|  | Where was this service provided? | 1 = Health Post  2 = Health Center  3 = Government Hospital  4 = Private Hospital/clinic  5 = At home  88 = Other (specify) ____________________ | | |  |
|  | Which was the provider who gave you drug for intestinal worms the first time?  Put 99 if not known | 1 = Skilled Health care provider  2 = FLW  3 = HEW  4 = TBA  5 = vCHW(HDA)  88 = Others (specify) ____________  99 = Don’t know | | |  |
|  | Were you given an injection in the arm to prevent the you and your baby from getting tetanus? | 1 = Yes  2 = No | | | **2🡪540** |
|  | Where was this service provided? | 1 = Health Post  2 = Health Center  3 = Government Hospital  4 = Private Hospital/clinic  5 = At outreach sites  88 = Other (specify) ____________________ | | |  |
|  | Which was the provider who gave you TT vaccine the first time?  Put 99 if not known | 1= skilled Health care provider  2 = FLW  3 = HEW  4 = TBA  5 = vCHW(HDA)  88 = Others (specify) ____________  99 = Don’t know | | |  |
|  | How many times did you get a tetanus injection? | [________________] | | |  |
|  | Did you receive HIV counseling and HIV testing? | 1 = Yes  2 = No | | | **2🡪543** |
|  | Where was this service provided? | 1 = Health Post  2 = Health Center  3 = Government Hospital  4 = Private Hospital/clinic  5 = At home  88 = Other (specify) ____________________ | | |  |
|  | Which was the provider who gave you information on HIV and did HIV testing the first time?  Put 99 if not known | 1= skilled Health care provider  2 = FLW  3 = HEW  4 = TBA  5 = vCHW(HDA)  88 = Others (specify) ____________  99 = Don’t know | | |  |
|  | Did you receive STI information and STI testing including syphilis? | 1 = Yes  2 = No | | | **2🡪547** |
|  | Where was this service provided? | 1 = Health Post  2 = Health Center  3 = Government Hospital  4 = Private Hospital/clinic  5 = At home  88 = Other (specify) ____________________ | | |  |
|  | Which was the provider who gave you information on STI and did STI testing the first time?  Put 99 if not known | 1= skilled Health care provider  2 = FLW  3 = HEW  4 = TBA  5 = vCHW(HDA)  88 = Others (specify) ____________  99 = Don’t know | | |  |
|  | If you were told that you have STI, did you receive treatment? | 1 = Yes  2 = No | | |  |
|  | Did you receive information on nutrition? | 1 = Yes  2 = No | | | **2🡪550** |
|  | Where was this service provided? | 1 = Health Post  2 = Health Center  3 = Government Hospital  4 = Private Hospital/clinic  5 = At home  88 = Other (specify) ____________________ | | |  |
|  | Which was the provider who gave you information on nutrition the first time?  Put 99 if not known | 1= skilled Health care provider  2 = FLW  3 = HEW  4 = TBA  5 = vCHW(HDA)  88 = Others (specify) ____________  99 = Don’t know | | |  |
|  | In your recent pregnancy, how was your feeding practice as compared to the amount you took before? | 1 = Much Less  2 = Somewhat less  3 = About the same  4 = More than usual  99 = Don’t Know | | |  |
|  | Were you advised on birth preparedness and complication readiness? | 1 = Yes  2 = No | | | **2🡪554** |
|  | Where was this service provided? | 1 = Health Post  2 = Health Center  3 = Government Hospital  4 = Private Hospital/clinic  5 = At home  88 = Other (specify) ____________________ | | |  |
|  | Which was the provider who advised you on birth preparedness and complication readiness the first time?  Put 99 if not known | 1= skilled Health care provider  2 = FLW  3 = HEW  4 = TBA  5 = vCHW(HDA)  88 = Others (specify) ____________  99 = Don’t know | | |  |
|  | During any of your ANC care visit were you told about the danger signs that occur during pregnancy, delivery and postnatal periods? | 1 = Yes  2 = No | | | **2🡪558** |
|  | Which danger signs of pregnancy were you told about?  (Don’t read the list)  (Multiple answer possible)  Probe once- what else | 1 = Vaginal bleeding during pregnancy  2 = High fever  3 = Severe headache  4 = Swelling of hands and face  5 = High blood pressure  6 = Severe lower abdominal pain  7 = Anemia /low blood /light headedness/ dizziness, pallor palmer & conjunctive  8 = Malaria( fever, shivering, headache sign)  9 = Blurred vision  10 = Convulsion  88 = Others(Specify)______________________  99 = Don’t know | | |  |
|  | Which danger signs of labor and birth were you told about?  (Don’t read the list)  (Multiple answer possible)  Probe once- what else | 1 = High fever  2 = Severe headache / Blurred vision  3 = Labor >12 hours  4 = Convulsions/fit/eclampsia  5 = Mal-presentation (baby’s hand, foot, buttock, or cord before head)  6 = Premature rupture of membrane (labor started 6 hours after the membrane has ruptured)  7 = Cord around baby’s neck  8 = Reduced/absent fetal movement  9 = Bleeding during labor  10 = Excessive bleeding after birth  11 = Ruptured uterus  88 = Others (Specify:_________________  99 = Don't know | | |  |
|  | Which danger signs of postnatal period were you told about?  (Don’t read the list)  (Multiple answer possible)  Probe once- what else | 1 = High fever  2 = Severe headache / Blurred vision  3 = Severe pain in calf (with or without swelling)  4 = Severe lower abdominal pain  5 = Foul smelling discharge  6 = Retained placenta  7 = Convulsions/fit/eclampsia  8 = Excessive bleeding after birth  9 = postpartum psychosis  88 = Others (Specify:_________________  99 = Don't know | | |  |
|  | Can you tell me the components of birth preparedness and complication readiness plan  (Don’t read the list)  (Multiple answer possible)  Probe once- what else | 1 = Financial  2 = Transport  3 = Nutrition and sufficient food  4 = Identify birth attendant  5 = Identify birth place  6 = Identify blood donor  7 = Clean cloth  8 = Cover to deliver on  9 = Gloves  10 = Soap  11 = Cotton Gauze  12 = New Razor blade  13 = Sterilized scissors  14 = Sterilized thread  88 = Others (specify)__________________ | | |  |
|  | Did you make any preparations for your delivery? | 1 = Yes  2 = No | | | **2🡪561** |
|  | If yes **Q. 559:** What preparations did you make for your delivery?  **Multiple response** | 1 = Financial  2 = Transport  3 = Nutrition and sufficient food  4 = Identify birth attendant  5 = Identify birth place  6 = Identify blood donor  7 = Clean cloth  8 = Cover to deliver on  9 = Gloves  10 = Soap  11 = Cotton Gauze  12 = New Razor blade  13 = Sterilized scissors  14 = Sterilized thread  88 = Others (specify)__________________ | | |  |
|  | During your last pregnancy have you attended a pregnant women’s conference/Forum? | 1 = Yes  2 = No | | | **2🡪566** |
|  | If yes **Q. 561**, how many times did you attend pregnant women’s forum in your last pregnancy? | ____________________ | | |  |
|  | What was discussed in the pregnant women’s forum?  **Probe but do not read out the list. Select all that apply**. | 1 = Birth preparedness  2 = Importance of ANC  3 = Institutional delivery  4 = Importance of PNC checks  5 = Seeking newborn care  88 = Others (specify) __________________ | | |  |
|  | Who informed you about the pregnant women’s forum? | 1 =HEW  2 = FLW  3 = TBA  4 = vCHW/HDA  5 = Other (Specify)_____________________ | | |  |
|  | Where did the pregnant women conference take place? | 1 = Gote/kebele meeting place  2 = health post  3 = health center/Hospital  4 = Other (specify) ______________________ | | |  |
|  | Can you tell us whether or not you were satisfied with the care you received while you were pregnant?  Do not read list of options | 1 = Yes was satisfied  2 = No was not satisfied  3 = Neither satisfied nor dissatisfied | | |  |
|  | If yes **Q. 566**, then what was the level of satisfaction  Read both options | 1 = Fully satisfied-  2 = Somewhat satisfied- | | |  |
|  | **If No Q. 566,** then what was the level of dissatisfaction  Read both options | 1 = Fully dissatisfied  2 = Somewhat dissatisfied | | |  |
|  | **Interviewer**  If the interviewee has reported having facility based care for ANC for this birth, ask the following questions  When having a pregnancy related medical examination at a health facility, did you experience any of the following with someone?  **Select all that apply** | **No** | **Variables** | **1=Y**  **2=N** |  |
|  |  |  | Encourage you to ask questions about delivery |  |  |
|  |  |  | Give you the choice for choosing any specific option (date and mode of delivery, etc.) |  |  |
|  |  |  | Unnecessarily motivating you for having C/S |  |  |
|  |  |  | Give explanation on the procedure s/he is going to use (e.g. normal delivery or C/S) |  |  |
|  |  |  | Maintain privacy while examining you (e.g. placed a separator before examination) |  |  |
|  |  |  | Use non-dignified language (for example passing insulting and/or degrading statements about you/family/community/ethnicity) during examination |  |  |
|  |  |  | The care provider deny to provide you services because you belong to any specific ethnic group |  |  |
|  |  |  | Use harsh tone or shouted on your during examination |  |  |
|  |  |  | Use abusive language with you during examination |  |  |
|  |  |  | Threaten you e.g. if you do not cooperate I will do so and so |  |  |
|  |  |  | Leave you abandoned during examination |  |  |
|  |  |  | Share results/diagnosis of medical reports with you during examination |  |  |
|  |  |  | Share the results when other could easily hear |  |  |
|  |  |  | Assure you that your medical information/ records will be kept confidential |  |  |
|  |  |  | Deny you providing any specific services (that you asked for) due to lack of money |  |  |

| **Section VI: Delivery Care** | | | | | |
| --- | --- | --- | --- | --- | --- |
| **Interviewer**: Now I have some questions to ask you about what happened to you during and immediately after delivery for CHILD NAME/PREGNANCY NUMBER | | | | | |
|  | Where did you give birth? | 1 = Own Home  2 = Other’s home  3 = Public hospital  4 = Health centers  5 = Health post  6 = Others(government)  7 = NGO’s health facilities  8 = Private hospital/clinic  9 = Others (private)  88 = Others (specify) ___________________ | | |  |
|  | If delivered at Home, Why? | 1 = Not necessary to give birth at health facility  2 = Not customary  3 = Husband/family did not allow  4 = No female provider at facility  5 = Don’t trust health providers  6 = Facility was closed  7 = Too far/no transportation  8 = Cost too much  9 = Poor quality service  10 = Tradition/religious reason  11 = No privacy  12 = Fear of abuse/disrespect  13 = Not aware of the service  88 = Other (specify) __________________ | | |  |
|  | If delivered in health post, health center or hospital, why? | 1 = Always delivered at a health facility  2 = Due to existing complications  3 = Referred by HDA/HEW  4 = Advised at pregnant women’s forum  5 = Taken to health facility due to prolonged labor or delivery related risks  6 = Convenient  7 = Affordable (free)  88 = Others, Specify |­­­­­_________________| | | |  |
|  | Who was the primary person (attendant) that assisted you with the delivery? | 1 = Skilled health care provider  2 = FLW  3 = HEW  4 = TBA  5 = vCHW  6 = Family/Friend/relatives  7 = No one was present  88. Other (Specify: ­­­­­­­­­­­­­­____________________) | | |  |
|  | Who else was present at the delivery? | 1 = skilled health care provider  2 = FLW  3 = HEW  4 = TBA  5 = vCHW  6 = Family/Friend/relatives  7 = No one was present  88. Other (Specify: ­­­­­­­­­­­­­­________________________) | | |  |
|  | Was your recent newborn delivered by caesarean section? | 1 = Yes  2 = No | | |  |
|  | Was the newborn/baby born alive? | 1 = Yes  2 = No | | | **2 🡪 609** |
|  | **If yes Q. 606**, Is your baby alive now? | 1 = Yes  2 = No | | | **1 🡪 609** |
|  | **If no Q. 607,** at what age did your child died? | [_________] days  or  [__________] months | | |  |
|  | When you gave birth, did the main attendant assisting you wash her/his hands with soap before the delivery? | 1 = Yes  2 = No  99 = Don’t know | | |  |
|  | When you gave birth, did the main attendant assisting you wear gloves during delivery? | 1 = Yes  2 = No  99 = Don’t know | | |  |
|  | When you gave birth, did the delivery take place on a clean place? (Clean surface: clean space or carpet or mat) | 1 = Yes  2 = No  99 = Don’t know | | |  |
|  | When you gave birth, did the main attendant used clean birth things? | 1 = Yes  2 = No  99 = Don’t know | | |  |
|  | Were you given a drug called misoprostol to help prevent/control bleeding? | 1 = Yes  2 = No  99 = Don’t know | | |  |
|  | **Interviewer:** If the woman gave birth at a health facility, ask her  After giving birth, for how long did you stay at the health facility in total? | [_____________] hours    or  [_____________] days | | |  |
|  | During the delivery of your recent child, did you experience any of the following? | 1 = High fever  2 = Severe headache / Blurred vision  3 = Labor >12 hours  4 = Convulsions/fit/eclampsia  5 = Mal-presentation (baby’s hand, foot, buttock, or cord before head)  6 = Premature rupture of membrane (labor started 6 hours after the membrane has ruptured)  7 = Cord around baby’s neck  8 = Reduced/absent fetal movement  9 = Bleeding during labor  10 = Excessive bleeding after birth  11 = Ruptured uterus  88 = Others (Specify:_________________  99 = Don't know | | |  |
|  | During delivery were you advised to go to a higher health facility to get special care? | 1 = Yes  2 = No | | | **2 🡪 621** |
|  | What was the reason for which you were referred? | 1 = Due to existence of one or more danger signs  2 = Due to lack of equipment/space  3 = Due to lack of trained human resources  4 = Other (specify) _______________________ | | |  |
|  | When you were referred to a higher health facility, did you go to the higher health facility? | 1 = Yes  2 = No | | | **1 🡪 621** |
|  | **If No Q. 618,** why? | 1 = Facility was too far  2 = Cost too much money  3 = Don’t like going to different facility  4 = No permission to go  5 = Don’t like the quality of care provided  6 = Other (specify) ______________________ | | |  |
|  | Was ambulance made available for you for that referral? | 1 = Yes  2 = No | | |  |
|  | Can you tell us whether or not you were satisfied with the delivery care you received?  Do not read list of options | 1 = Yes was satisfied  2 = No was not satisfied  3 = Neither satisfied nor dissatisfied | | | **2 🡪 623** |
|  | **If yes Q. 621,** then what was the level of satisfaction  Read both options | 1 = Fully satisfied  2 = Somewhat satisfied - | | |  |
|  | **If No Q. 621,** , then what was the level of dissatisfaction  Read both options | 1 = Fully dissatisfied  2 = Somewhat dissatisfied | | |  |
|  | During a birth at a health facility, did you experience any of the following with someone?  **Select all that apply** | **No** | **Variables** | **1=Y**  **2=N** |  |
|  |  |  | Regularly explain and inform you about the progress or procedure being performed |  |  |
|  |  |  | Cover you while taking to the delivery room |  |  |
|  |  |  | Delay in providing care after a decision has been made e.g. caesarean section |  |  |
|  |  |  | Use abusive language with you during the delivery |  |  |
|  |  |  | Leave you abandoned during the delivery |  |  |
|  |  |  | Perform any procedure without your consent or informing you (like caesarean sections, blood transfusion, sterilization etc.) |  |  |
|  |  |  | Ignore you while asking pain relief/medication during delivery |  |  |
|  |  |  | Use harsh tone or shouted on you during the delivery |  |  |
|  |  |  | Slap you during the delivery |  |  |
|  |  |  | Pinch you irritably during the delivery |  |  |
|  |  |  | Beat you during the delivery |  |  |
|  |  |  | Push you badly to change your position during the delivery |  |  |
|  | Soon after giving birth at a facility, did you experience any of the following with someone?  **Select all that apply** | **SN** | **Variables** | **1=Y**  **2=N** |  |
|  |  |  | Encourage you to ask questions post-delivery |  |  |
|  |  |  | Cover you after the delivery |  |  |
|  |  |  | Ignore you while asking pain relief /medication after the delivery |  |  |
|  |  |  | Leave you abandoned immediately after the delivery |  |  |
|  |  |  | Use harsh tone or shouted on you after the delivery |  |  |
|  |  |  | Use abusive language with you after the delivery |  |  |
|  |  |  | Ask you to clean delivery couches post-delivery |  |  |
|  |  |  | Ask you to clean dirty bathroom/toilets post-delivery |  |  |
|  |  |  | Detain for non/partial-payment (e.g. keep you or your child in custody for full payment) |  |  |
|  | Did you know where to go to lodge a complaint | 1 = Yes  2 = No | | |  |

| **Section VII: Postnatal Care (PNC)** | | | |
| --- | --- | --- | --- |
| **Interviewer:** Now I want to ask you about any postnatal checks you had for your own health after that birth | | | |
|  | Did anyone check on your own health within 6 weeks of giving birth? | 1 = Yes  2 = No | **2 🡪 800** |
|  | How many days after giving birth did you have your first health check?  Clarify that this is a health check for the mother | [__________] days  99 = Don’t know |  |
|  | Where did the first check take place? | 1 = Health Post  2 = Health Center  3 = Government Hospital  4 = Private Hospital/clinic  5 = At home  Other (specify) ____________________ |  |
|  | By whom? | 1 = Skilled health care provider  2 = FLW  3 = HEW  4 = TBA  5 = vCHW(HDA)  88 = Others (specify) ____________  99 = Don’t know |  |
|  | Was a second visit conducted? | 1 = Yes  2 = No |  |
|  | How many days after giving birth did you have your second health check?  Clarify that this is a health check for the mother. | [__________] days  99 = Don’t know |  |
|  | Where did the checking take place? | 1 = Health Post  2 = Health Center  3 = Government Hospital  4 = Private Hospital/clinic  5 = At home  Other (specify) ____________________ |  |
|  | By whom? | 1 = Skilled health care provider  2 = FLW  3 = HEW  4 = TBA  5 = vCHW(HDA)  88 = Others (specify) ____________  99 = Don’t know |  |
|  | Was a third visit conducted? | 1 = Yes  2 = No |  |
|  | How many days after giving birth did you have your second health check?  Clarify that this is a health check for the mother. | [__________] days  99 = Don’t know |  |
|  | Where did the checking take place? | 1 = Health Post  2 = Health Center  3 = Government Hospital  4 = Private Hospital/clinic  5 = At home  Other (specify) ____________________ |  |
|  | By whom? | 1 = Skilled health care provider  2 = FLW  3 = HEW  4 = TBA  5 = vCHW(HDA)  88 = Others (specify) ____________  99 = Don’t know |  |

| During visits after giving birth, what was done to check your health? **Read List out loud**  **Mark all that apply**  **If the visit did not happen at specified time, leave that column of answers blank** | | | | | | |
| --- | --- | --- | --- | --- | --- | --- |
|  | **For each: 1 = Yes 2 = No** | | | | | |
|  | **(Q. #)** | **0-2 days** | **(Q. #)** | **3-7 days** | **(Q. #)** | **8-42 days** |
| Checked breasts |  |  |  |  |  |  |
| Checked uterus |  |  |  |  |  |  |
| Advised breast feeding |  |  |  |  |  |  |
| Orientation about danger signs |  |  |  |  |  |  |
| Educated on family planning |  |  |  |  |  |  |
| Information given on nutrition |  |  |  |  |  |  |
| Referred to a health facility |  |  |  |  |  |  |
| Measured Blood Pressure |  |  |  |  |  |  |
| Checked/treated birth-related wound (if applicable) |  |  |  |  |  |  |
| Other |  |  |  |  |  |  |
| Specify ___________ |  | | | | | |

|  | Can you tell us whether or not you were satisfied with the post-natal care you received?  Interviewer: explain that this is care for the mother, not the newborn. Do not read list of options | 1 = Yes was satisfied  2 = No was not satisfied  3 = Neither satisfied nor dissatisfied |  |
| --- | --- | --- | --- |
|  | **If Yes Q. 742,** then what was the level of satisfaction  Read both options | 1 = Fully satisfied  2 = Somewhat satisfied |  |
|  | **If No Q. 742,** then what was the level of dissatisfaction  Read both options | 1 = Fully dissatisfied  2 = Somewhat dissatisfied |  |

| **VIII. Immediate newborn care** | | | |
| --- | --- | --- | --- |
| **Interviewer: if the pregnancy outcome in the pregnancy history table was (stillbirth), end the interview here**  **Now I have some questions about what happened to [CHILD NAME] at birth and immediately afterwards.** | | | |
|  | **Interviewer:** What is the pregnancy ID number of [CHILD NAME]? | **[____________] ID number** | **From Table** |
|  | Was this a single birth? | 1 =Yes  2 =No |  |
|  | Where was [CHILD NAME] placed immediately after delivery? | 1 = Alone/on the floor  2 = On the mother’s Belly/ chest  3 = Beside the mother  4 = With someone else  5 = Other  6 = Don’t know |  |
|  | Did anyone do any of the following to [CHILD NAME] immediately after birth? | 1 = Dry  2 = Wrap/swaddle  3 = Check the breathing  4 = Back rub |  |
|  | How long after [CHILD NAME] was born was s/he dried/wiped?  Check for time after the baby was born, not time after the placenta came out. | [____________] minutes,  99 = Don’t know. |  |
|  | How long after [CHILD NAME] was born was s/he wrapped in a cloth?  Check for time after the baby was born, not time after the placenta came out. | [____________] minutes,  99 = Don’t know. |  |
|  | Did [CHILD NAME] have any difficulty crying/ breathing at birth? | 1 = Yes  2 = No  99 = Don’t know | **2 🡪 808** |
|  | **If yes Q. 806,** did anyone do any of the following to [CHILD NAME] to help the breathing? | 1 = Rubbing/stimulating  2 = Mouth-to-mouth resuscitation  3 = Ambu-bag resuscitation |  |
|  | Did you breastfeed [CHILD NAME]? | 1 = Yes  2 = No | **2 🡪 811** |
|  | **If yes Q. 808**, how long after birth did you first put [CHILD NAME] to the breast? | 1 = In the first hour  2 = After one hour but during  the first day  3 = After the first day of life |  |
|  | What did you do with the first milk? | 1 = Squeeze out and throw  2 = Squeeze out and give to the baby  88 = Other (specify)___________________  99 = Do not know |  |
|  | **If No Q. 808,** what did you mainly give [CHILD NAME], other than breast milk? | 1 = Water  2 = Milk (other than breast milk)  3 = Plain water  4 = Sugar or glucose water  5 = Fruit juice  6 = Infant Formula  7 = Tea/infusion  8 = Fresh butter  88 = Other, specify_________________ |  |
|  | And, why did you provide other drinks besides your breast milk? | 1 = My breast milk is not enough  2 = I do not stay with NAME throughout the day  3 = Advised by friends or relatives to do so  4 = Tradition or culture  5 = Other |  |
|  | Did you give only breast milk [CHILD NAME] during the first 28 days of life? | 1 = Yes  2 = No |  |
|  | Frequency of breast feeding? | 1 = 1-7 times a day  2 = 8-12 times a day  3 = More than 12 times a day  4 = Never  99 = Don’t know |  |
|  | Did [CHILD NAME] receive TTC eye ointment soon after delivery? | 1 = Yes  2 = No  99 = Don’t know |  |
|  | What was used to tie the cord? | 1 = New string/thread  2 = Boiled string/thread  3 = Any string/thread  4 = Clamp  5 = Nothing  6 = Don’t know  7 = Other |  |
|  | What was used to cut the cord? | 1 = new razor blade  2 = any razor blade  3 = sterilized scissors  4 = any scissors  5 = don’t know  6 = other |  |
|  | Did you keep the cord exposed and dry? | 1 = Yes  2 = No |  |
|  | Was anything applied to the cord after tying and cutting? | 1 = Yes  2 = No | **2 🡪 821** |
|  | **If yes Q.819:** What was applied to the cord just after cutting the cord?  **Do not prompt, select all that apply** | 1 = Butter  2 = ash  3 = Ointment (non-medicinal)  4 = Animal dung  5 = Oil  6 = Cold water  88 = Other (Specify) ___________ |  |
|  | Was an antiseptic applied to the cord? | 1 = Yes  2 = No  99 = Don’t know | **2 🡪 825** |
|  | **If yes Q. 821**, was this Chlorhexidine? | 1 = Yes  2 = No  99 = Don’t know | **2 🡪 825** |
|  | **If yes Q. 822**, for how many days was Chlorhexidine applied? | [_________] days  99 = not known |  |
|  | **If yes Q. 823**, how many times per day was Chlorhexidine applied? | [________]# of times applied per day  99 = Don’t know |  |
|  | Did [CHILD NAME] receive vitamin K? | 1 = Yes  2 = No  99 = Don’t know |  |
|  | Was [CHILD NAME] weighed at birth? | 1 = Yes  2 = No  99 = Don’t know | **2 🡪 830** |
|  | **If Yes Q. 826**: how much did [CHILD NAME] weigh at birth? | [_________] weight in grams  99= Don’t know |  |
|  | Is the baby LBW (<2.5Kg) or premature baby? | 1 = Yes  2 = No  99 = Don’t know |  |
|  | In the first week of life, did you hold [CHILD NAME] skin to skin against your chest during the daytime and night-time? | 1 = Yes,  2 = No  99 = Don’t know |  |
|  | In the first week of life, did you sleep with [CHILD NAME] against you at night, or did you lay him/her alone on the bed or elsewhere? | 1 = Slept with mother  2 = Baby slept alone  3 = Baby slept with another  Person |  |
|  | When [CHILD NAME] was born, how soon did you bath him/her? | 1 = In the first hour  2 = After one hour  3 = After one day |  |
|  | If in the first hour: After how many minutes would you say? | [________] minutes.  99 = Don’t know |  |
|  | If after one hour: After how many hours would you say? | [________] hours  99 = Don’t know | If it is 1 -2, enter 1hr, If it is >2, enter 2hr |
|  | If after one day: After how many days would you say?  E.g. if response is between day one and two, enter 1. | [_______] full days.  99 = Don’t know |  |
|  | Immediately after [CHILD NAME] was born, did any one check on his/her health?  Probe for checks done at the place of birth on the same day as birth | 1 = Yes  2 = No |  |
|  | Did you receive counseling on newborn care? | 1 = Yes  2 = No | **2 🡪 838** |
|  | **If Yes Q. 836,** in what topics you received counseling? | 1 = Breast feeding  2 = Recognizing danger signs  3 = Cleanliness  4 = Cord care  5 = Immunization  88 = Other (specify) _____________ |  |
|  | In the first 6 weeks after [CHILD NAME] was born, did any one check on his/her health?  Probe for checks done at the place of birth on the same day as birth, and checks after. | 1 = Yes  2 = No | **2 🡪 890** |
|  | **If Yes Q. 838**, how many days after birth was [CHILD NAME]’s first health check? | [___________] days  99 = Don’t know |  |
|  | **If Yes Q. 838,** where did the health checks on [CHILD NAME] take place? | 1 = Health Post  2 = Health Center  3 = Government Hospital  4 = Private Hospital/clinic  5 = At home  Other (specify) _________________ |  |
|  | **If Yes Q. 838**, who checked on the health of [CHILD NAME]?  Probe for most qualified person | 1 = Skilled health care provider  2 = FLW  3 = HEW  4 = TBA  5 = vCHW(HDA)  88 = Others (specify) ___________  99 = Don’t know |  |
|  | Was a second check conducted? | 1 = Yes  2 = No | **2 🡪 854** |
|  | **If Yes Q. 842**, how many days after birth was [CHILD NAME]’s second health check? | [___________] days  99 = Don’t know |  |
|  | **If Yes Q. 842**, Where did the health checks on [CHILD NAME] take place? | 1 = Health Post  2 = Health Center  3 = Government Hospital  4 = Private Hospital/clinic  5 = At home  Other (specify) _________________ |  |
|  | **If Yes Q. 842**, Who checked on the health of [CHILD NAME]?  Probe for most qualified person | 1 = skilled health care provider  2 = FLW  3 = HEW  4 = TBA  5 = vCHW(HDA)  88 = Others (specify) ___________  99 = Don’t know |  |
|  | Was a third check conducted? | 1 = Yes  2 = No | **2 🡪 854** |
|  | **If Yes Q. 846,** how many days after birth was [CHILD NAME]’s third health check? | [___________] days  99 = Don’t know |  |
|  | **If Yes Q. 846,** Where did the health checks on [CHILD NAME] take place? | 1 = Health Post  2 = Health Center  3 = Government Hospital  4 = Private Hospital/clinic  5 = At home  Other (specify) _________________ |  |
|  | **If Yes Q. 846**, Who checked on the health of [CHILD NAME]?  Probe for most qualified person | 1 = skilled health care provider  2 = FLW  3 = HEW  4 = TBA  5 = vCHW(HDA)  88 = Others (specify) ___________  99 = Don’t know |  |
|  | Was a fourth check conducted? | 1 = Yes  2 = No | **2 🡪 854** |
|  | **If Yes Q. 850,** how many days after birth was [CHILD NAME]’s forth health check? | [___________] days  99 = Don’t know |  |
|  | **If Yes Q. 850,** Where did the checking take place? | 1 = Health Post  2 = Health Center  3 = Government Hospital  4 = Private Hospital/clinic  5 = At home  Other (specify) _________________ |  |
|  | **If Yes Q. 850,** Who checked on the health of [CHILD NAME]?  Probe for most qualified person | 1 = skilled health care provider  2 = FLW  3 = HEW  4 = TBA  5 = vCHW(HDA)  88 = Others (specify) ___________  99 = Don’t know |  |

| During visits after giving birth, what was done to check your health? **Read List out loud**  **Mark all that apply**  **If the visit did not happen at specified time, leave that column of answers blank** | | | | | | |
| --- | --- | --- | --- | --- | --- | --- |
|  | **For each: 1 = Yes 2 = No** | | | | | |
|  | **(Q. #)** | **0-2 days** | **(Q. #)** | **3-7 days** | **(Q. #)** | **8-42 days** |
| Generally examined /looked at baby’s body |  |  |  |  |  |  |
| Weighed baby |  |  |  |  |  |  |
| Checked cord |  |  |  |  |  |  |
| Advised breastfeeding |  |  |  |  |  |  |
| Observed breastfeeding |  |  |  |  |  |  |
| Advised skin-to-skin contact/warmth |  |  |  |  |  |  |
| Checked baby for danger signs (including sepsis) |  |  |  |  |  |  |
| Educated on danger signs |  |  |  |  |  |  |
| Referred to a health facility for danger sign |  |  |  |  |  |  |
| Provided information on washing hands before touching baby |  |  |  |  |  |  |
| Advised keeping the cord clean |  |  |  |  |  |  |
| Advised not to bathe the baby within 24 hours after birth |  |  |  |  |  |  |

|  | Can you tell us whether or not you were satisfied with the immediate newborn care?  Do not read list of options | 1 = Yes was satisfied  2 = No was not satisfied  3 = Neither satisfied nor dissatisfied |  |
| --- | --- | --- | --- |
|  | **If Yes Q.890,** then what was the level of satisfaction  Read both options | 1 = Fully satisfied  2 = Somewhat satisfied |  |
|  | **If No Q.890,** then what was the level of dissatisfaction  Read both options | 1 = Fully satisfied  2 = Somewhat satisfied |  |
|  | The last time you gave birth, did you keep your newborn at home for several days or weeks without taking the baby out? | 1 = Yes  2 = No | **2 🡪 895** |
|  | **If Yes Q.893**, what is the number of days that you kept your newborn in the house? | [___________] days  99 = Don’t know |  |
|  | The last time you gave birth, how many days passed before you had visitors come to your house to see the baby? This includes visitors for any reason: health care workers, extended family, or friends. | [___________] days  99 = Don’t know |  |
|  | The last time you gave birth, how many days passed before someone other than you had physical contact with the baby? Physical contact means any kind of touching, even if the person did not hold the baby. | [___________] days  99 = Don’t know |  |

| **IX. Care of Sick Newborns** | | | | | | | | | | | | | | | | | | | | |
| --- | --- | --- | --- | --- | --- | --- | --- | --- | --- | --- | --- | --- | --- | --- | --- | --- | --- | --- | --- | --- |
|  | | | | **Interviewer** – stop to check: was the baby born after January 2008 Ethiopian calendar?  If yes, continue with interview.  If no, end interview at this point | | | | | | | | 1 = Yes  2 = No | | | | | | 2= End interview | | |
| **Interviewer*:*** *Now I want to talk to you about any sickness [CHILD NAME] experienced in the first two months of life* | | | | | | | | | | | | | | | | | | | | |
|  | | | Has [CHILD NAME] ever been sick during first two months of life? | | | | | | | | | 1 = Yes  2 = No | | | | | |  | | |
| Can I just check, has [CHILD NAME] ever had any of the following symptoms during the first two months of life?  Read list of symptoms out loud | | | | | | | | | | | | | | | | | | | | |
| **Enter 1 if Yes and continue along row**  **Enter 2 if No and go to next symptom (row)**  **If no symptoms, go to Section 10** | | | | | | | **Age at first episode**  **Enter age in number of days** | | **Was care sought for?**  **1 = Yes**  **2 = No** | | **If YES, where did you seek care from?**  **1 = Gov. HP**  **2 = Gov. HC**  **3 = Gov. Hosp.**  **4 = Private hosp./HDA**  **5 = Traditional Healers**  **6 = Pharmacy** | | | | **If care was sought, who cared for (CHILD NAME?)**  **1 = HO, Nurse, MD,**  **2 = pharmacist**  **3 = FLW**  **4 = HEW**  **5 = TBA**  **6 = vCHW(HDA)**  **88 = Others**  **99 = Don’t know** | | **If care was not sought, why not?**  **1 = Expected him/her to get better**  **2 = Health facility too far**  **3 = Cost of treatment too expensive**  **4 = Don’t trust the facility**  **5 = Family member didn’t allow**  **6 = Community advisor advised against it**  **7 = Other** | | | |
| Reduced feeding | | | | |  |  |  |  |  |  |  | | |  |  |  |  | | |  |
| Unable to suck | | | | |  |  |  |  |  |  |  | | |  |  |  |  | | |  |
| Child was underweight | | | | |  |  |  |  |  |  |  | | |  |  |  |  | | |  |
| Difficult or fast breathing | | | | |  |  |  |  |  |  |  | | |  |  |  |  | | |  |
| Chest in-drawing | | | | |  |  |  |  |  |  |  | | |  |  |  |  | | |  |
| Unusually hot or cold | | | | |  |  |  |  |  |  |  | | |  |  |  |  | | |  |
| Less active than usual | | | | |  |  |  |  |  |  |  | | |  |  |  |  | | |  |
| Yellow palms/ soles/eyes | | | | |  |  |  |  |  |  |  | | |  |  |  |  | | |  |
| Had diarrhea | | | | |  |  |  |  |  |  |  | | |  |  |  |  | | |  |
| Convulsions | | | | |  |  |  |  |  |  |  | | |  |  |  |  | | |  |
| Skin pustules | | | | |  |  |  |  |  |  |  | | |  |  |  |  | | |  |
| Cord red or draining pus | | | | |  |  |  |  |  |  |  | | |  |  |  |  | | |  |
| Other (specify) | | | | |  |  |  |  |  |  |  | | |  |  |  |  | | |  |
| Interviewer: fill in questions 980 - 989 only if in the above table the interviewee mentioned that care was sought out for any one of the symptoms mentioned | | | | | | | | | | | | | | | | | | | | |
|  | If any of the above symptoms occurred  After how many days did you seek care the first time? | | | | | | | | | | | | [___________] days  if first day of illness, write 0;  If possible use the medical record to confirm  99 if care not sought | | | | | |  | |
|  | Was the [CHILD NAME] diagnosed with very severe disease by a health worker?  Interviewer: list the signs for very sever disease for the mother | | | | | | | | | | | | 1 = Yes  2 = No | | | | | | **2 🡪 1000** | |
|  | **If Yes Q. 981, w**as [CHILD NAME] prescribed medicine for his/her illness? | | | | | | | | | | | | 1 = Yes  2 = No | | | | | | **2 🡪 985** | |
|  | Did [CHILD NAME] receive 7 consecutive days of gentamycin injection? | | | | | | | | | | | | 1 = Yes  2 = No | | | | | |  | |
|  | Did [CHILD NAME] receive 7 consecutive days of Amoxicillin?  Interviewer: explain Amoxicillin as an oral tablet that is dissolved and taken as a liquid. | | | | | | | | | | | | 1 = Yes  2 = No | | | | | |  | |
|  | At any time during the illness, did [CHILD NAME] take any drugs for the illness? | | | | | | | | | | | | 1 = Yes  2 = No | | | | | |  | |
|  | | Was [CHILD NAME] managed with expressed breast milk? | | | | | | | | | | | 1 = Yes  2 = No | | | | | |  | |
|  | | Can you tell us whether or not you were satisfied with the care for the sick newborn?  Do not read list of options | | | | | | | | | | | 1 = Yes was satisfied  2 = No was not satisfied  3 = Neither satisfied nor dissatisfied | | | | | |  | |
|  | | **If Yes Q. 987**, then what was the level of satisfaction  Read both options | | | | | | | | | | | 1 = Fully satisfied  2 = Somewhat satisfied | | | | | |  | |
|  | | **If No Q. 987**, then what was the level of dissatisfaction  Read both options | | | | | | | | | | | 1 = Fully dissatisfied  2 = Somewhat dissatisfied | | | | | |  | |

| **Section X: Children no longer alive (and died before reaching 28 days)** | | | | | |
| --- | --- | --- | --- | --- | --- |
| Interviewer: if child is no longer alive and died before reaching 28 days, ask the following questions around symptoms, care-seeking, and cause of death  *I understand that it is not easy to talk about children who have died and so please do let me know if you need time to answer the questions. This information is important and will allow the government can develop programs to improve children's health.* | | | | | |
| Which symptoms did you observe in (CHILD NAME’S) immediately before death?  Select all that apply | | For each: 1 = Yes 2 = No | | | |
|  |  |  | Difficult or fast breathing | |  |
|  |  |  | Chest in-drawing | |  |
|  |  |  | Unusually hot or cold | |  |
|  |  |  | Less active than usual | |  |
|  |  |  | Yellow palms/soles/eyes | |  |
|  |  |  | Had diarrhea | |  |
|  |  |  | Convulsions | |  |
|  |  |  | Yellow palms/soles/eyes | |  |
|  |  |  | Skin pustules | |  |
|  |  |  | Cord red or draining puss | |  |
|  |  |  | Other (specify) | |  |
|  | If CHILD NAME died due to an illness, was care sought for that illness? | | | 1 = Yes  2 = No–go to end of questionnaire |  |
|  | If yes, who provided that care?  Put 9 if not known | | | 1 = skilled health care provider  2 = FLW  3 = HEW  4 = Pharmacist  5 = TBA  6 = vCHW(HDA)  88 = Others (specify) ___________  99 = Don’t know |  |
|  | If care was sought before [CHILD NAME] died, what was the cause of death diagnosed by the health providers | | | 1 = Pneumonia  2 = Diarrhea  3 = Severe infections  4 = Other (Specify)_____________  99 = Don’t know |  |

**That is the end of our interview. Thank you very much for taking the time to answer these questions.**

Time at end of interview: ____:____

**THANK YOU!!**

**For oversee only!**

*I approve that this questionnaire was duly filled:*

**Name**  **Position/responsibility** **Signature and Date**

1. ____________________ __________________________ ______________________
2. ____________________ __________________________ ______________________
3. ____________________ __________________________ ______________________
